# The Circadian Clock Is Sustained in the Thyroid Gland of VIP Receptor 2 Deficient Mice

**DOI:** 10.3389/fendo.2021.737581

**Published:** 2021-09-01

**Authors:** Birgitte Georg, Jan Fahrenkrug, Henrik L. Jørgensen, Jens Hannibal

**Affiliations:** ^1^Department of Clinical Biochemistry, Bispebjerg and Frederiksberg Hospital, Faculty of Health Sciences, University of Copenhagen, Copenhagen, Denmark; ^2^Department of Clinical Biochemistry, Amager and Hvidovre Hospital, Copenhagen, Denmark; ^3^Department of Clinical Medicine, University of Copenhagen, Copenhagen, Denmark

**Keywords:** thyroid gland, VPAC2 receptor, knockout mice, circadian rhythm, clock genes

## Abstract

VIP/VPAC2-receptor signaling is crucial for functioning of the circadian clock in the suprachiasmatic nucleus (SCN) since the lack results in disrupted synchrony between SCN cells and altered locomotor activity, body temperature, hormone secretion and heart rhythm. Endocrine glands, including the thyroid, show daily oscillations in clock gene expression and hormone secretion, and SCN projections target neurosecretory hypothalamic thyroid-stimulating hormone (TSH)-releasing hormone cells. The aim of the study was to gain knowledge of mechanisms important for regulation of the thyroid clock by evaluating the impact of VIP/VPAC2-receptor signaling. Quantifications of mRNAs of three clock genes (*Per1*, *Per2* and *Bmal1*) in thyroids of wild type (WT) and VPAC2-receptor deficient mice were done by qPCR. Tissues were taken every 4^th^ h during 24-h 12:12 light-dark (LD) and constant darkness (DD) periods, both genders were used. PER1 immunoreactivity was visualized on sections of both WT and VPAC2 lacking mice during a LD cycle. Finally, TSH and the thyroid hormone T4 levels were measured in the sera by commercial ELISAs. During LD, rhythmic expression of all three mRNA was found in both the WT and knockout animals. In VPAC2-receptor knockout animals, the amplitudes were approximately halved compared to the ones in the WT mice. In the WT, *Per1* mRNA peaked around “sunset”, *Per2* mRNA followed with approximately 2 h, while *Bmal1* mRNA was in antiphase with *Per1*. In the VPAC2 knockout mice, the phases of the mRNAs were advanced approximately 5 h compared to the WT. During DD, the phases of all the mRNAs were identical to the ones found during LD in both groups of mice. PER1 immunoreactivity was delayed compared to its mRNA and peaked during the night in follicular cells of both the thyroid and parathyroid glands in the WT animals. In WT animals, TSH was high around the transition to darkness compared to light-on, while T4 did not change during the 24 h cycle. In conclusion, sustained and identical rhythms (phases and amplitudes) of three clock genes were found in VPAC2 deficient mice during LD and DD suggesting high degree of independence of the thyroid clock from the master SCN clock.

## Introduction

Circadian rhythms are 24 h endogenous daily oscillations in physiology and behaviour such as metabolism, blood pressure, body temperature, blood hormone levels, sleep and locomotor activity securing synchrony with the geophysical surroundings. The internal circadian timing system consists of a master pacemaker located in the hypothalamic suprachiasmatic nucleus (SCN) and multiple peripheral clocks located in cells of every organ of the body (1–4). The 24 h oscillation in each cell is the result of an autoregulatory transcription-translation feedback loop of clock genes. The activator proteins BMAL1 and CLOCK heterodimerize and activate the transcription of the clock genes *Per1* and *Per2* and the cryptochromes (*Cry1* and *Cry2*) that act as repressors of CLOCK-BMAL1 (2, 4, 5). The master SCN clock is adjusted daily primarily by photic cues transmitted from the retina *via* a monosynaptic nervous pathway, the retinohypothalamic tract (3, 4, 6). The circadian rhythm of peripheral organs is regulated both by the SCN through blood borne and neuronal signals and directly through e.g. metabolic signals and physical activity. In addition, there is interplay between the circadian rhythms in the peripheral organs through hormones which also affects the SCN clock (1, 2, 4, 7).

The neuropeptide Vasoactive Intestinal Polypeptide (VIP) and its receptor VPAC2 are both highly expressed in the SCN (8, 9), and VIPergic signaling *via* the VPAC2 receptor plays an essential role in the control of circadian activity in the SCN promoting rhythmicity and synchronizing SCN neurons (8–13). Mice lacking either VIP or the VPAC2 receptor thus display disrupted circadian rhythmicity of physiology and activity in addition to attenuated expression of clock genes (*Per1, Per2* and *Bmal1)* in the SCN (14). During diurnal light/dark conditions, VPAC2 knockout (KO) animals show stable nocturnal locomotor activity which contrasts constant darkness (DD), where VPAC2 KO mice become arrhythmic in both activity and clock gene expression (10, 13, 15). Although the activity rhythms of VPAC2 KO are similar to that of wild-type (WT) animals during LD cycles, the 24 h rhythmicity in core body temperature, metabolism, food intake, clock gene expression in peripheral organs and corticosterone secretion in VIP/VPAC_2_ KOs are attenuated and/or advanced compared to the ones in wildtype animals (9, 11, 13, 15–18).

Thyroid hormones are important for metabolism, development and growth. In addition, they play an essential role for seasonal adaption of e.g. reproduction. The synthesis of thyroid hormones is regulated by the hypothalamic-pituitary-thyroid (HPT) axis which is under circadian control (7, 19), although serum TSH and thyroid hormone levels in addition are influenced by multiple other factors (20).

We have previously shown that the thyroid follicular cells of the rat contain a circadian clock which is independent of pituitary inputs (21), but the interplay between the thyroid and the central SCN clock is still under explored. As the VPAC2 KO mice have a defective central circadian clock, these mice were used to gain more insight to the regulation of the thyroid clock by examining the diurnal and circadian expression of thyroid core clock genes (*Per1, Per2* and *Bmal1*) and serum levels of TSH and T4.

## Materials and Methods

### Animals

The VPAC2 WT and VPAC2 KO mice (22) used in the study were bred on location from heterozygote animals. The two groups of animals were equal in number and included both genders. The mice were housed in 12h:12h LD (Zeitgeber time ZT0 ~ lights on and ZT12 ~ lights off) with *ad libitum* access to food and water. Animals were treated according to the principles of Laboratory Animal Care (Law on Animal Experiments in Denmark, LBK NR474, May 15, 2014) and Dyreforsoegstilsynet, Ministry of Food, Agriculture and Fisheries of Denmark, who issued the license number 2017/15-0201-01364 to Jens Hannibal thereby approving the study.

### Experiments

Animals (4 females and 4 males of each genotype for each time-point) used for analysis of clock gene expression and hormone measurements during LD were entrained to 12h:12h LD light cycles for 2 - 3 weeks before decapitation and organ removal at each of the following time points: ZT4, ZT8, ZT12, ZT16, ZT20 and ZT24 (ZT0 = light-on; ZT12 = light-off. Animals used for immunohistochemistry (three in total at each time point) were kept under similar conditions until they at time points matching those used for mRNA quantifications were anesthetized and transcardially perfused for 3 min with heparin (15,000 IE/L phosphate buffered saline, pH 7.2) followed by Stefanini’s fixative (2% PFA, 15% picric acid in 0.1 M phosphate buffer) for 15 min. Anesthesia were done by subcutaneous injection of hypnorm and midazolam (0.1 mL per 10 g body weight; 0.08 g/L fentanyl citrate, 2.5 g/L fluanisone, 1.25 g/L midazolam).

Animals used in DD experiments (same number as for LD) were as the ones taken in LD entrained to 12h:12h LD cycles for at least 2 – 3 weeks. Hereafter, light was not turned on at ZT0, and during the second day after transfer into continuous darkness, the animals were decapitated in dim red light at time points corresponding to circadian time (CT) 4, 8, 12, 15, 20 and 24.

### Tissue Preparation

After decapitation, blood was collected for serum preparation, and thyroid glands were removed on wet ice as tissue blocks also containing the trachea. Tissues were quick frozen on dry ice, and both sera and tissue blocks were stored at -80°C until use. Before RNA preparation, the thyroid glands (including the parathyroid glands) were dissected on ice from the frozen tissue blocks. Thyroid glands used for immunohistochemistry were cryoprotected in 30% sucrose after dissection and stored at -80°C until use.

### RNA Extraction, cDNA Synthesis and qPCR

Total RNA from dissected glands was prepared by the guanidinium thiocyanate–phenol–chloroform extraction method (23). cDNA was made using High-Capacity cDNA Archive Kit (Thermo Fisher Scientific) using 1 µg total RNA in a total reaction volume of 100 µL. The tissues were from the animals used in a study of clock gene expression in the adrenal gland (15).

Quantifications of *Per1, Per2, Bmal1*, thyroid peroxidase (*Tpo*) and parathyroid hormone (*Pth*) mRNA were done by qPCR using a StepOnePlus instrument and TaqMan-based chemistry (Thermo Fisher Scientific). Quantification of β2-microglobulin (*B2m*) mRNA used as internal control and *Per1* and *Bmal1* mRNA including standard curves were as the ones previously described (15). Quantifications of *Per2*, *Tpo and Pth* mRNA were done using TaqMan Gene Expression Assay Mm00478099_m1, Mm00456355_m1 and Mm01271501_m1 (Thermo Fisher Scientific), respectively. The standard curves for these assays were made using pooled thyroid RNA as template but otherwise as previously described (24). qPCR was run in 20 µL containing cDNA from 20 ng total RNA using TaqMan Universal PCR Master Mix containing AmpErase7UNG (Thermo Fisher Scientific). *B2m* internal control reactions were run in separate wells on each plate detecting the target genes, and all samples, standards and the non-template negative controls were made in duplicates. The StepOne Software v. 2.3 (Thermo Fisher Scientific) was used to calculate concentrations in arbitrary units of target (*Per1, Per2, Bmal1 Tpo* or *Pth*) and *B2m*, and the amount of target gene was normalized with the *B2m* mRNA amount from the same plate.

### Immunohistochemistry

Thyroid glands from fixated animals were cut on a cryostat in 12 µm thick sections. PER1 immunohistochemistry was performed as previously described (14), using PER1 antiserum raised and characterized in own laboratory (25) and 4′,6′-diamidino-2-phenylindole (DAPI) was used for counterstaining. Fluorescence images were obtained using an iMIC confocal microscope (TILL Photonics, FEI, Germany) with the appropriate filter settings for detection of Alexa 488 and DAPI. Images were edited for contrast and brightness by Adobe Photoshop (Adobe Systems) and combined into plates using Adobe Illustrator (Adobe Systems).

### Hormone ELISAs

Serum concentrations of TSHb were measured by Mouse Thyrotropin beta (TSHb) ELISA kit (abx254595, Abbexa Ldt., Cambridge, UK), and T4 was determined using DetectX THYROXINE (T_4_) Enzyme Immunoassay Kit (K050, Arbor Assays, Ann Arbor, USA).

### Statistical Analysis

All concentrations are presented as means ± standard error of mean (SEM). The method for cosinor rhythmometry as described by Nelson and coworkers (26) was used to determine diurnal/circadian rhythmicity in clock gene mRNAs and hormone concentrations by fitting the data to a combined cosine and sine function: Per = M + k1COS(2πt/24) + k2SIN(2πt/24). Substituting COS(2πt/24) = C and SIN(2πt/24) = Z gives the expression: Per = M + k1C + k2Z. The model fit was then tested using the general linear model procedure in the SAS statistical software package (SAS Enterprise Guide 7.1). For analysis of differences in hormone levels Kruskal-Wallis test followed by Dunn’s multiple comparison test of selected columns was performed. P < 0.05 was considered statistically significant.

## Results

### Oscillations of Clock Gene mRNA in Thyroids of WT and VPAC2-KO Mice During LD

**Figure 1:**
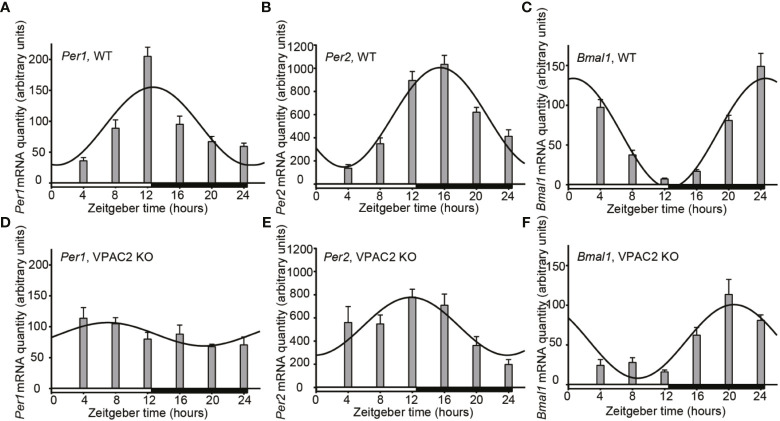
Daily oscillation of clock gene mRNA in thyroid glands from wild-type (WT) and VPAC2 receptor knockout (KO) mice. RNA from thyroid glands of animals during 12:12 h light-dark cycles was extracted, and the amounts of *Per1* **(A, D)**, *Per2* **(B, E)**, and *Bmal1* **(C, F)** mRNA were quantified by qPCR. β2-microglobulin amounts were used for normalisation. The results are given as mean arbitrary units ± SEM, n = 8, and wild-type and KO animals are shown in **(A–C)** and D – F, respectively. In both groups of mice, the expression of all three clock genes displayed significant oscillations as a function of the 24 h LD-cycle (P < 0.001 for **A–C, E, F**, and P < 0.05 for **D**). The curves show the 24 h cosinor fittings of the data. The white and black bars at the bottom of each graph represent the period of light and darkness during the tissue sampling period.

We compared the thyroid clock of WT and VPAC2 deficient mice by quantifying the mRNAs encoded by clock genes *Per1, Per2* and *Bmal1* during a 12h:12h LD cycle by qPCR. The results shown in **Figure 1** revealed that all three mRNAs exhibit significant cyclic oscillation as a function of the 24h cycle (**Table 1**). In the thyroid of WT animals, *Per1* mRNA level was low around “dawn” (ZT0) and peaked approximately at “sun set” (ZT12), the phase of *Per2* mRNA was delayed 3 h compared to *Per1* leading to maximal amount at early night, while the phase of *Bmal1* mRNA was in antiphase with *Per1* peaking at light-on (**Figures 1A–C**). In thyroid glands of VPAC2 KO animals, the 24 h rhythmic expression of all three clock genes was preserved with the same mean levels but with significantly reduced amplitudes and/or phase advances compared to WT. Thus, we found that the phase advances were approximately 5 h while the amplitudes halved (**Figures 1D–F**, **Tables 1** and **2**).

**Table 1:** Parameters of 24-h rhythmometric analysis of *Per1, Per2* and *Bmal1* mRNA expression in thyroid glands of wildtype (WT) and VPAC2 receptor knockout mice (VPAC2 KO) during light-darkness.

LD	WT			VPAC2 KO		
	*Per1*	*Per2*	*Bmal1*	*Per1*	*Per2*	*Bmal1*
T_max_ (ZT)	12:40	15:28	00:34	06:58	11:51	20:39
T_min_ (ZT)	00:40	03:28	12:34	18:58	23:51	08:39
Amplitude	123	858	138	38	499	93
Mesor/mean	93	577	65	88	528	54
Significance (P)	<0.0001	<0.0001	<0.0001	<0.05	<0.0001	<0.0001

**Table 2:** Significance of differences in rhythmometric parameters from **Tables 1** and **3** between wildtype (WT) and VPAC2 receptor knockout mice (VPAC2 KO) during both light-darkness (LD) and constant darkness (DD).

WT/VPAC2 KO	LD			DD		
	*Per1*	*Per2*	*Bmal1*	*Per1*	*Per2*	*Bmal1*	
Amplitude/phase	<0.0001	0.001	<0.00001	0.0001	<0.00001	<0.000001	
Mesor/mean	N.S.*	N.S.	N.S.	N.S.	N.S.	<0.001	

*N.S., Not significant.

### Sustained Clock Gene mRNA Oscillation During DD in Thyroids of Both WT and VPAC2-KO Mice

**Figure 2:**
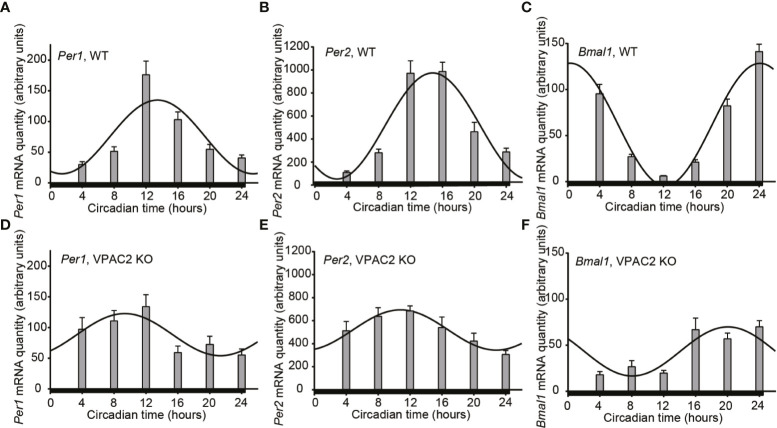
Circadian oscillation of clock gene expression in thyroid glands from wild-type (WT) and VPAC2 receptor knockout (KO) mice. RNA from thyroid glands of animals during the second cycle of constant darkness was extracted, and the amounts of *Per1* **(A, D)**, *Per2* **(B, E)**, and *Bmal1* **(C, F)** mRNA were quantified by qPCR. β2-microglobulin amounts were used for normalisation. The results are given as mean arbitrary units ± SEM, n = 7 – 8, and WT and KO animals are shown in **(A–F)** respectively. In both groups of mice, the expression of all three clock genes displayed significant oscillation as a function of the 24 h (P < 0.001 for **A–C, E, F**, and P < 0.01 for **D**). The curves show the 24 h cosinor fittings of the data. The black bar at the bottom of each graph represents the darkness during the tissue sampling period.

Circadian rhythmicity of many physiological parameters is lost in VPAC2 KO animals during DD conditions (10, 13, 15). We therefore analyzed the expression of the three clock genes (*Per1, Per2* and *Bmal1*) in thyroid glands during the second day of constant darkness. In WT animals, comparable oscillations of all three mRNAs (*Per1, Per2* or *Bmal1*) were found as during LD (**Figures 2A–C**, **Table 3**). In thyroid glands of VPAC2 KO animals, the expression of the clock genes also continued to be rhythmically expressed as during LD, only the amplitude (and/or phase) of *Bmal1* was found to be decreased in VPAC2-KO animals during DD compared to LD (**Figure 2**, **Tables 3** and **4**). As during LD, the oscillations of the three clock genes showed significant difference in amplitude and/or phase between the two groups of animals (**Table 2**). The means of *Per1* and *Per2* mRNA were similar in the two groups, while *Bmal1* mRNA was found to be reduced in VPAC2 KO compared to WT animals (**Table 2**).

**Table 3:** Parameters of 24-h rhythmometric analysis of *Per1, Per2* and *Bmal1* mRNA expression in thyroid glands of wildtype (WT) and VPAC2 receptor knockout mice (VPAC2 KO) during constant darkness.

DD	WT			VPAC2 KO		
	*Per1*	*Per2*	*Bmal1*	*Per1*	*Per2*	*Bmal1*
T_max_ (CT)	13:25	14:47	00:19	09:21	10:49	20:03
T_min_ (CT)	01:25	02:47	12:19	21:21	22:49	08:03
Amplitude	122	919	132	69	351	53
Mesor/mean	76	488	62	88	519	43
Significance (P)	<0.0001	<0.0001	<0.0001	<0.01	<0.001	<0.0001

**Table 4:** Significance of differences in rhythmometric parameters from **Tables 1** and **3** between light-darkness and constant darkness in wildtype (WT) and VPAC2 receptor knockout mice (VPAC2 KO), respectively.

LD/DD	WT			VPAC2 KO		
	*Per1*	*Per2*	*Bmal1*	*Per1*	*Per2*	*Bmal1*
Amplitude/phase	N.S.*	N.S.	N.S.	N.S.	N.S.	<0.05
Mesor/mean	<0.05	<0.05	N.S.	N.S.	N.S.	N.S.

*N.S., Not significant.

### PER1 Oscillates in Follicular Cells of Both the Thyroid and Parathyroid Gland

**Figure 3:**
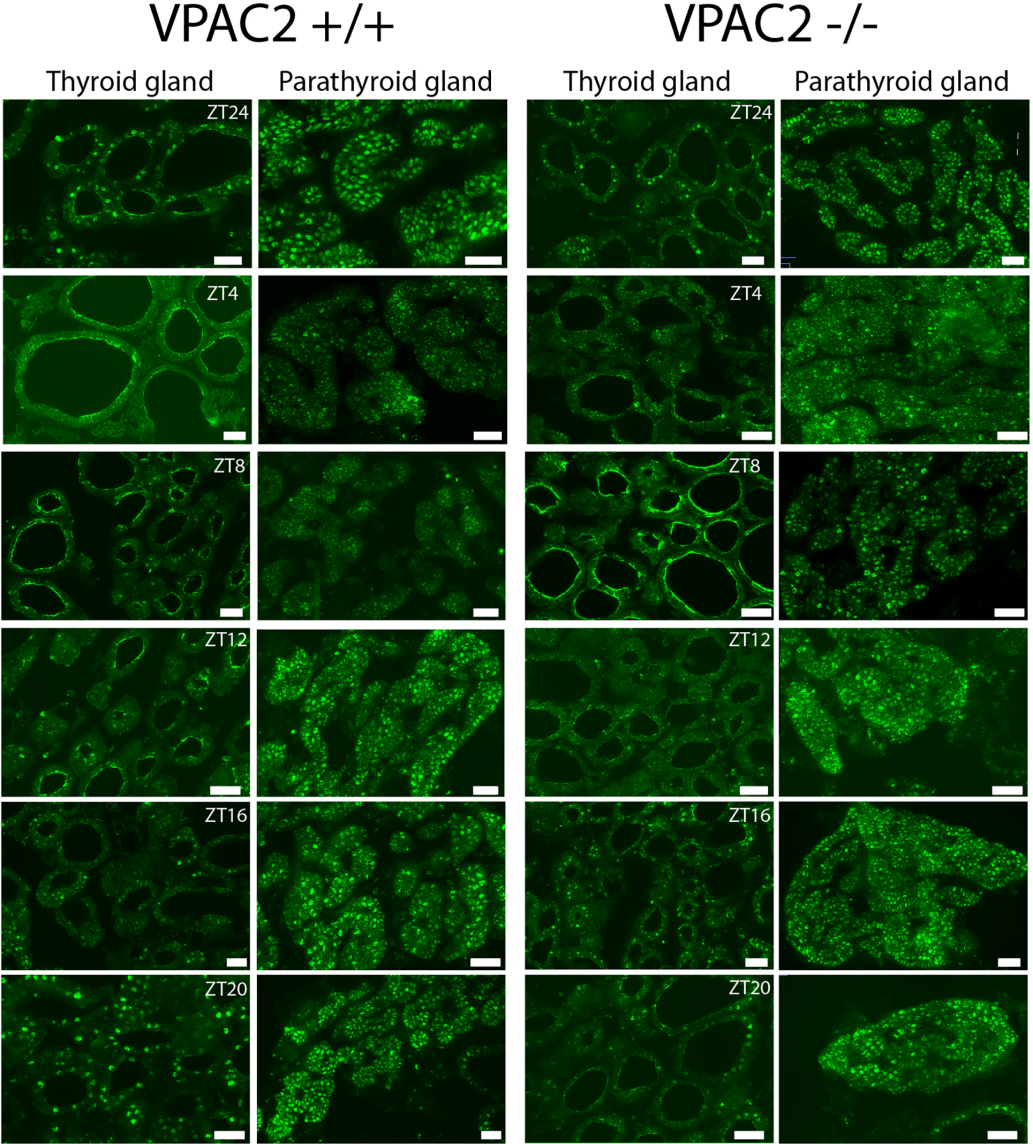
Immunohistochemical staining of PER1 in the thyroid and parathyroid glands of VPAC2 wild-type and deficient mice during a 12:12 h light/dark periods. The two left rows show staining of the glands from wild-type and the two right rows staining of the knockout animals. PER1 is present in follicular cells in both the thyroid and parathyroid glands of both wildtype and VPAC2 deficient mice. Scale bars: 50 µm.

Immunohistochemistry was used to evaluate the cellular localisation of clock components (PER1) in the thyroid and parathyroid glands during a 24 h cycle, which also made it possible to evaluate whether the phase of the clock was identical in the two. **Figure 3** shows PER1 expression in follicular cells of both the thyroid and parathyroid gland in both WT and VPAC2 KO animals during 12:12 LD cycles. In WT animals, the level of immunoreactive PER1 oscillated during the daily cycle with peak expression in thyroid follicular epithelial at late night (ZT20 – ZT24, **Figure 3**, row 1). The oscillation of PER1 in VPAC2 KO appeared a little earlier around ZT16 (**Figure 3**, row 3), and immunoreactivity seemed lower compared to the WT animals. Similar phase of PER1 positive cells was found in the parathyroid as the thyroid gland in WT animals (**Figure 3**, row 2). As in the thyroid gland, oscillation was more difficult to in reveal in the parathyroid from VPAC2 KO animals but as for the thyroid gland seemed to appear earlier in these compared to the WT animal (**Figure 3**, row 4).

### The Daily Profiles of Circulating TSH and T4

**Figure 4:**
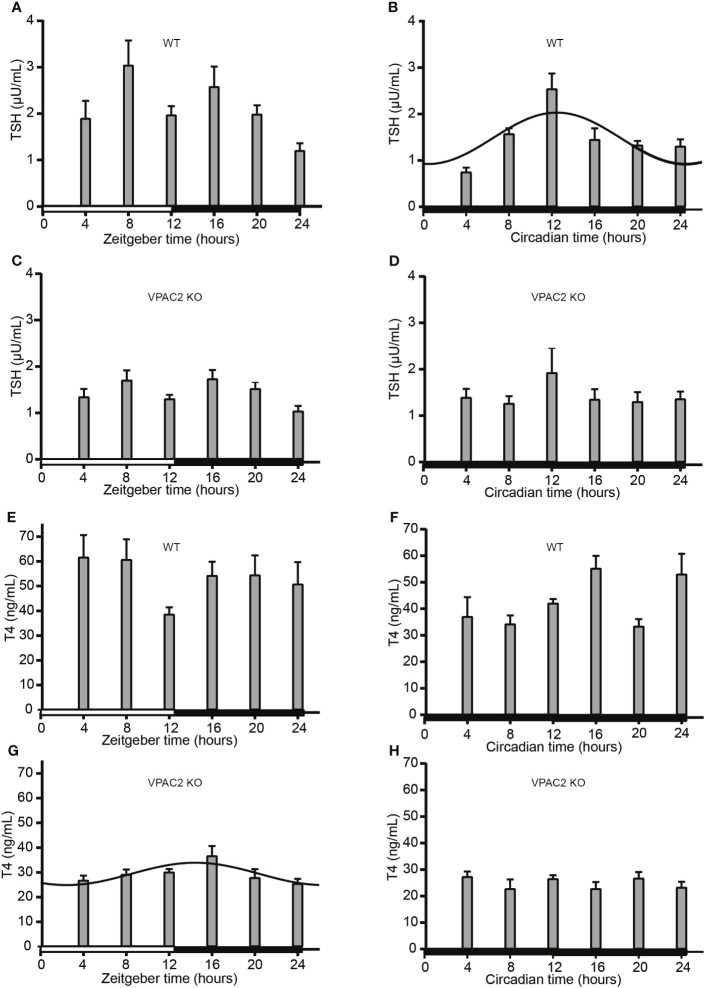
Serum concentrations of TSH **(A–D)** and T4 **(E–H)** in wild-type (WT) **(A, B, E, F)** and VPAC2 receptor knockout (KO) mice **(C, D, G, H)** during a 12:12 h light/dark period **(A, C, E, G)** and constant darkness **(B, D, F, H)**. Commercial ELISA kits were used for the quantification of hormone concentrations in serum. TSH **(A–D)** in sera from WT animals **(A)** and T4 **(G)** in sera from KO mice fitted 24 h cosinor rhythmicity. In WT animals, the TSH concentration was significantly higher at ZT/CT12 than ZT/CT24.

To evaluate the interconnection between TSH, thyroid hormone levels and the thyroid clock, we also analyzed the circulating levels during 24 h periods in LD and DD. The results are shown in **Figure 4**. In WT animals, serum TSH changed during the daily cycle during both LD (**Figure 4A**) being significantly higher in LD at ZT8 (P = 0.001) and ZT 16 (P = 0.01) than ZT24. In DD, a statistically significant 24 h oscillation was found (P = 0.001) with maximal expression at CT12:30 (**Figure 4B**). In VPAC2 deficient mice (**Figures 4C, D**), the same tendency was seen, although to lesser extent and thus we did not reveal significant difference in concentration between the time-points. Serum T4 (**Figures 4E–H**) in WT animals did neither exhibit 24 h oscillation nor variation in amount during the daily cycle LD (**Figure 4E**), while T4 in serum from VPAC2-KO animals seemed to fit 24 h oscillation (**Figure 4G**) although no differences in the concentration was revealed by Kruskal-Wallis test. During DD, higher concentrations of T4 were found at early night than during the day in WT but not in VPAC2 KO animals (**Figures 4F, H**).

## Discussion

VPAC2 KO mice were used to evaluate regulatory aspects of the thyroid clock, as the intercellular communication in the SCN of these mice is hampered due to the lack of VIP signaling. In addition to the lost intercellular synchrony of SCN cells, lack of VIP/VPAC2 signaling results in deficient gating of light mediated responses (27, 28). An important function of the master clock in the SCN is to receive input and by neuronal, humoral and other signaling pathways to ensure both synchrony between the various peripheral clocks and entrainment to the daily LD cycle and thereby securing optimal coherence of the entire body to the surroundings (29, 30). The SCN cells expressing VPAC2 are not only important for maintenance of SCN rhythmicity but also for SCN output responses such as activity (31, 32). The importance of VIP/VPAC2 signaling in relation to circadian rhythmicity make VPAC2 KO animals well suited as a model for studies of the maintenance and regulation of peripheral clock function in organs such as the thyroid/parathyroid glands.

We analyzed the expression of three clock genes (*Per1, Per2* and *Bmal1*) and found the same amplitude and phase for all three in both LD and DD in the WT and VPAC2 deficient mice, respectively. For the WT animals, the maximal expressions of the mRNAs were in strict accordance with the ones identified for a number of other peripheral tissues (33). The advanced phase and decreased amplitude of clock gene expression in the thyroid gland of VPAC2 KO animals during LD also accords with previous findings of the clock gene expression in the liver, adrenal gland and heart in addition to physiological responses such as body temperature, hormone levels, intraocular pressure, heart rate, feeding and locomotor activity observed in VPAC2 and/or VIP deficient mice (13, 15, 16, 18, 34, 35). Although, we found difference in both amplitude and phase of clock gene expression between WT and VPAC2 KO animals, similar mean levels of clock gene mRNA were found indicating it is primarily the oscillating profile and not the amount of clock proteins which is compromised in the VPAC2 KO animals. Whether rhythmicity is abolished during DD in VPAC2 and VIP deficient animals seems, however, to be dependent on the response of the specific organ involved. In the present study, we show continuation of the oscillation of thyroid clock gene expression, which is opposed to the findings in the adrenal gland (15) and in the striatum and the motor cortex of the brain where the oscillation of clock gene expression is abolished during DD in VPAC2 deficient animals (10). In addition, many physiological responses such as corticosterone, heart rate, activity, and body temperature also become arhythmic in VPAC2 and VIP deficient mice during constant conditions (13, 15, 34, 35). However, the oscillation of clock gene expression in the liver and heart of VPAC2 deficient animals continues with advanced phase during DD (16), comparable to the findings in the thyroid gland reported here. The variable responses between peripheral organs in VPAC2 KO animals after transfer to DD is probably due both to differences in the dependence of these organs on the SCN, the signaling pathway from the SCN to the specific organ and the robustness of the internal clock of the specific organ. In our strain of VPAC2 deficient mice all animals become arrhythmic from the first day in constant darkness (13), while other strains of VPAC2 KO mice have more variable arrhythmic behavior during constant darkness (10). An explanation for this difference between strains and the advanced phase in clock gene expression and physiology still needs further studies to be understood.

By immunohistochemistry, we found oscillation of clock protein (PER1) in follicular cells of both the thyroid and the parathyroid glands with maximal expression appearing at the same time in the two glands, and with the maximal expression delayed compared to the mRNA. Because both the thyroid and parathyroid glands seemed to be in similar phases, we see no drawbacks in analyzing mRNA samples containing both. The major component of the samples was of thyroid origin, while parathyroid tissue only contributed with a minor part.

The thyroid gland receives SCN input both *via* the HPT, as SCN directly innervates TRH neurons in the paraventricular nucleus, and by sympathetic and parasympathetic autonomic input, the latter probably being involved in the regulation of the sensitivity of the thyroid gland to different stimuli (36). The same study showed, that SCN lesions affected the daily profile of thyroid hormones although TSH was unaffected. We have previously shown, that hypophysectomy of rats does not abolish the thyroid circadian clock suggesting that the HPT-axis is not the primary route for setting this clock (21). Concomitantly, we showed that a functional thyroid clock was unable to account for daily oscillations of thyroid hormones. These studies illustrate a complex relationship between the thyroid hormone regulation and the circadian clock. Different results of levels and oscillation of TSH and the thyroid hormones T4 and T3 during the daily cycles have been reported dependent on the animal species, gender and study parameters (37). In humans, TSH is rhythmically expressed being high during early night and low at day (7), while in rats the profile is opposite (21, 36, 38–40). The data on mice are very sparse. Our present results only demonstrated circadian oscillation of TSH in WT mice during DD with maximal expression around the transition between light and darkness. During LD, WT animals had high circulating TSH levels at ZT8 (late day) and ZT16 (early night) compared to low level at the transition between night and day (ZT24). During LD, the TSH profile of VPAC2 deficient mice mimicked that of WT but it was more blunted. A similar biphasic profile has been shown in some rat studies (36, 41). In addition to the circadian TSH rhythm, ultradian TSH rhythms exist and on top of that TSH is affected by sleep and food intake as well by various hormones including melatonin (7, 20, 42, 43). Variable results have been obtained concerning diurnal expression of serum thyroid hormone levels in rats (21, 38, 39). Our results of serum T4 did not show robust 24 h oscillation and we did not find diurnal or circadian variation of the level of Tpo mRNA (data not shown). It is noteworthy here that nocturnal rodents have multiphasic sleep during light as opposed to the more consolidated sleep of humans during darkness (7), possibly explaining the difference in robustness in diurnal oscillations in TSH and thyroid hormones. In addition, many laboratory mice strains, including C57, contain mutations in the enzymes responsible for melatonin synthesis making them melatonin deficient (44). It will be interesting to see whether melatonin proficient mice have a more robust diurnal rhythm of TSH. It is clear from this study that although both TSH and thyroid hormones under some conditions exert diurnal and circadian oscillation, the regulation is complex and not primarily a result of a functional clock in the follicular cells of the gland. However, the robustness of the thyroid points toward another not yet understood function of the circadian clock in the thyroid gland.

We also found PER1 in follicular cells of the parathyroid gland showing that these cells harbor an oscillating clock. Daily variations in serum PTH have been shown in humans (45, 46) but not in rats (41). We also analyzed the for PTH mRNA in samples containing the parathyroid and thyroid glands in both groups of animals during LD and DD and we did not find any diurnal or circadian variations (data not shown) which accords with previous findings of PTH in rodents not showing diurnal variation. This could, however, also be due to the fact that the mRNA used for the analysis was derived from both thyroid and parathyroid tissue and that the parathyroid represented the minor part, thus being blunted by expression of the *B2m* internal control present in both.

In conclusion: We have demonstrated that the clock of the thyroid gland is circadian, advanced and blunted in VPAC2 deficient animals although these animals have defective synchronization of the SCN clock. The thyroid clock is however not correlated to the daily changes in hormone levels.

## Data Availability Statement

The original contributions presented in the study are included in the article/**Supplementary Material**. Further inquiries can be directed to the corresponding author.

## Ethics Statement

The animal study was reviewed and approved by Dyreforsoegstilsynet, Ministry of Food, Agriculture and Fisheries of Denmark.

## Author Contributions

BG, JF and JH designed the research. JF made first draft of the manuscript and contributed to design of all figures. BG performed the animal, ELISA and qPCR experiments and made the corresponding **Figures 1**, **2** and **4**. JH performed immunohistochemistry and made **Figure 3**. HJ and BG performed the statistics. BG and JH wrote the final manuscript. All authors contributed to the article and approved the submitted version.

## Funding

The work was supported by the Danish Biotechnology Center for Cellular Communication.

## Conflict of Interest

The authors declare that the research was conducted in the absence of any commercial or financial relationships that could be construed as a potential conflict of interest.

## Publisher’s Note

All claims expressed in this article are solely those of the authors and do not necessarily represent those of their affiliated organizations, or those of the publisher, the editors and the reviewers. Any product that may be evaluated in this article, or claim that may be made by its manufacturer, is not guaranteed or endorsed by the publisher.

## Abbreviations

CT, circadian time; DD, constant darkness; KO, knockout; LD, light-darkness; PTH: parathyroid hormone, VIP, vasoactive intestinal polypeptide; VPAC2, vasoactive intestinal peptide receptor 2; WT, wild-type; ZT, zeitgeber time.
